# Baseline plasma NfL, p‐tau217 and tau‐PET SUVr predict cognitive decline in non‐demented persons from a memory clinic

**DOI:** 10.1002/alz.088816

**Published:** 2025-01-09

**Authors:** Augusto J. Mendes, Federica Ribaldi, Aurelien Lathuiliere, Nicholas J. Ashton, Shorena Janelidze, Henrik Zetterberg, Max Scheffler, Frederic Assal, Valentina Garibotto, Kaj Blennow, Oskar Hansson, Giovanni B. Frisoni

**Affiliations:** ^1^ Laboratory of Neuroimaging of Aging (LANVIE), University of Geneva, Geneva Switzerland; ^2^ Geneva Memory Center, Department of Rehabilitation and Geriatrics, Geneva University Hospitals, Geneva Switzerland; ^3^ Department of Psychiatry and Neurochemistry, Institute of Neuroscience and Physiology, The Sahlgrenska Academy, University of Gothenburg, Mölndal, Gothenburg Sweden; ^4^ Clinical Memory Research Unit, Lund University, Lund Sweden; ^5^ Institute of Neuroscience and Physiology, Sahlgrenska Academy at the University of Gothenburg, Gothenburg Sweden; ^6^ Division of Radiology, Geneva University Hospitals, Geneva Switzerland; ^7^ Geneva University Hospitals, Geneva Switzerland; ^8^ Laboratory of Neuroimaging and Innovative Molecular Tracers (NIMTlab), Geneva University Neurocenter and Faculty of Medicine, University of Geneva, Geneve Switzerland; ^9^ Institute of Neuroscience and Physiology, Sahlgrenska Academy, Centre for Ageing and Health (AGECAP) at the University of Gothenburg, Gothenburg Sweden; ^10^ Clinical Memory Research Unit, Department of Clinical Sciences, Lund University, Lund Sweden; ^11^ University of Geneva, Geneva Switzerland

## Abstract

**Background:**

Plasma biomarkers have been increasingly studied in Alzheimer’s disease due to their potentially high accessibility, affordability, and low invasiveness. Recent studies have shown that baseline plasma levels are capable of predicting cognitive decline in cognitively unimpaired subjects (CU) and with mild cognitive impairment (MCI). Despite the fact that neuroimaging biomarkers are also strong predictors, it is still unclear how well they perform when compared to plasma biomarkers in predicting cognitive deterioration.

**Method:**

Our goal was to evaluate how plasma (i.e., p‐tau181, p‐tau231, p‐tau217, GFAP, and NfL) and traditional neuroimaging biomarkers (i.e., amyloid‐PET centiloid, tau‐PET global‐SUVr, and hippocampal volume) are able to predict cognitive decline in non‐demented individuals. A total of 100 subjects were included in this study, namely 67 MCI and 33 CU from the Geneva Memory Center cohort. The study applied linear mixed‐effect regression models to evaluate how baseline biomarker levels predict cognitive decline. Moreover, sample sizes for future AD clinical trials were calculated for a subset of amyloid‐positive subjects based on how well each biomarker could predict the disease.

**Result:**

Cognitive decline was only significantly predicted in the MCI subsample by baseline plasma NfL (β = ‐0.53, p = 0.02) p‐tau217 (β = ‐0.59, p = 0.009), and tau‐SUVR (β = ‐0.91, p = 0.002). Lastly, we have proven that the sample sizes for future treatment trials targeting AD can be reduced by up to 70% in individuals who exhibit both amyloid‐PET positivity by incorporating NfL as a requirement for inclusion. On the other hand, the inclusion of p‐tau217 might decrease amyloid‐positive sample sizes only by 10%.

**Conclusion:**

Tau‐SUVR was the best predictor of cognitive decline in the MCI stage, followed by plasma p‐tau217, and NfL. However, when the goal is to identify at‐risk subjects for future AD clinical trials with amyloid‐PET positivity in the inclusion criteria, the NfL seems to be the most appropriate biomarker to evaluate. This implies a strong association between amyloid‐PET and p‐tau217, whereas NfL may be more appropriate to identify individuals at‐risk for developing cognitive decline.

